# Comparison of Liver Transplant Wait-List Outcomes Among Patients With Hepatocellular Carcinoma With Public vs Private Medical Insurance

**DOI:** 10.1001/jamanetworkopen.2019.10326

**Published:** 2019-08-30

**Authors:** Liat Gutin, Francis Yao, Jennifer L. Dodge, Joshua Grab, Neil Mehta

**Affiliations:** 1 Department of Medicine, University of California, San Francisco; 2 Division of Transplant Surgery, Department of Surgery, University of California, San Francisco; 3 Division of Gastroenterology, Department of Medicine, University of California, San Francisco

## Abstract

**Importance:**

There are well-documented racial/ethnic and socioeconomic disparities in access to health care among patients with hepatocellular carcinoma (HCC); however, there are little data on the association of insurance type with liver transplant (LT) wait-list outcomes for patients with HCC.

**Objective:**

To examine LT wait-list outcomes for patients with HCC and public insurance compared with patients with private insurance.

**Design, Setting, and Participants:**

This single-center cohort study included 705 adult patients with HCC who had Model for End-Stage Liver Disease exceptions and were included on a waiting list for LT from January 1, 2010, to December 31, 2016. Patients with Kaiser Permanente medical insurance, other private medical insurance, or public medical insurance were included. Data analysis was conducted from May 2018 to October 2018.

**Main Outcomes and Measures:**

The main outcome was cumulative incidence of LT waiting list dropout within 2 years of waiting list enrollment (baseline). Secondary outcomes included competing-risks analysis to identify risk factors associated with wait-list outcomes.

**Results:**

Among 705 patients (median [interquartile range] age, 61 [57-65] years; 537 [76.2%] men) with HCC on an LT waiting list, 349 patients (49.5%) had Kaiser Permanente insurance, 157 patients (22.3%) had other private insurance, and 199 patients (28.2%) had public insurance. Median (interquartile range) follow-up was 13.2 (7.8-18.7) months. Tumor characteristics were similar among insurance types. The cumulative incidence of dropout owing to tumor progression or death within 2 years of baseline was 21.8% (95% CI, 17.2%-26.7%) among the Kaiser Permanente insurance group, 25.5% (95% CI, 18.6%-33.0%) among the other private insurance group, and 35.5% (95% CI, 28.3%-42.7%) among the public insurance group (*P* < .001). The cumulative incidence of LT within 2 years of baseline was 67.3% (95% CI, 61.2%-72.6%) among the Kaiser Permanente insurance group, 64.1% (95% CI, 55.2%-71.7%) among the other private insurance group, and 48.5% (95% CI, 40.4%-56.1%) among the public insurance group (*P* < .001). In competing-risks multivariable analysis compared with patients with Kaiser Permanente insurance, patients with public insurance were associated with increased risk of dropout (hazard ratio [HR], 1.69 [95% CI, 1.17-2.43]; *P* = .005), but patients with other private insurance were not (HR, 1.40 [95% CI, 0.94-2.08]; *P* = .10). Waiting list dropout was also significantly associated with an α-fetoprotein level 100 ng/mL or higher (HR, 2.8 [95% CI, 1.98-3.88]; *P* < .001), Model for End-Stage Liver Disease score at baseline (HR per point, 1.06 [95% CI, 1.03-1.09]; *P* < .001), and 3 or more lesions at baseline (HR vs 1 lesion of 2- to 3-cm diameter, 2.07 [95% CI, 1.27-3.37]; *P* = .004).

**Conclusions and Relevance:**

In this large cohort of patients with HCC on an LT waiting list, patients with public insurance were associated with worse wait-list outcomes compared with patients with Kaiser Permanente insurance or other private insurance, despite similar tumor-related characteristics at baseline. Improved health care coordination and delivery may be options to reduce these disparities.

## Key Points

**Question:**

Is type of insurance associated with liver transplant wait-list outcomes among patients with hepatocellular carcinoma?

**Findings:**

In this cohort study including 705 adult patients with hepatocellular carcinoma Model for End-Stage Liver Disease score exceptions at a single site initially wait-listed for liver transplant, 46.7% of patients with public medical insurance dropped out from the liver transplant wait-list compared with 28.7% of patients with Kaiser Permanente insurance and 33.8% of patients with other private medical insurance despite similar tumor-related characteristics.

**Meaning:**

Public insurance was associated with increased risk of waiting list dropout, and steps should be implemented to mitigate the increased risk of dropout among these patients.

## Teaser

This cohort study compares wait-list outcomes by insurance type for patients with hepatocellular carcinoma awaiting liver transplant.

## Introduction

Hepatocellular carcinoma (HCC) is an aggressive malignant neoplasm that is the fifth most common cancer worldwide and the third leading cause of cancer-related deaths.^1^ The incidence of HCC continues to increase in the United States, although a recent analysis showed that the rate of increase slowed between 2006 and 2011, likely owing to improved HCC primary prevention strategies, improvements in the treatment of viral hepatitis, and increased use of curative modalities.^2^ The only curative therapies for HCC are surgical, including tumor resection, liver transplant (LT), or local ablation.^3,4^

Despite these improvements in HCC incidence, there are well-documented socioeconomic and racial disparities in accessing care for patients with HCC. Black patients have incidence rates 2-fold that of white patients,^5^ and in a 2018 Surveillance, Epidemiology and End Results database study,^6^ the overall 5-year survival rate for patients with HCC was lower among black patients than any other racial group. Black patients were also the least likely to undergo curative therapy for HCC compared with white and Hispanic patients.^7^ Another study found that black patients were significantly less likely to undergo an LT despite similar severity of liver disease, tumor characteristics, and insurance status.^8^ Insurance type has also been shown to affect access to care for patients with HCC. Among US adults with HCC, patients who were uninsured or had Medicaid had more advanced tumor stage at diagnosis, lower rates of tumor-directed treatment, and lower overall survival.^9,10,11,12^ As more patients are using public insurance to cover the cost of LT since the Patient Protection and Affordable Care Act Medicaid expansion policy came into effect in 2014,^13,14^ these disparities are becoming increasingly concerning.

For patients with HCC with tumor burdens within the Milan criteria listed for LT, there are several challenges to ultimately receiving an LT, especially in regions with longer wait times,^15^ which can be further affected by socioeconomic status and insurance type. Transplant centers are often located in urban areas, and patients from rural areas may have to travel long distances frequently to attend appointments, undergo imaging and laboratory tests, or receive multiple bridging therapies. Furthermore, lower education levels and language barriers can make navigating the intricacies of an LT waiting list more challenging.

Although many studies have examined the associations of socioeconomic factors and insurance with the likelihood of receiving an LT,^9,10,11,12^ to our knowledge, there have been no studies specifically examining the association of insurance status and other socioeconomic variables with dropout from an LT waiting list among patients with HCC already wait-listed for LT. Given the many challenges that remain despite being successfully wait-listed for LT, we hypothesized that patients with public insurance in need of an LT would have worse wait-list outcomes than patients with private insurance, including Kaiser Permanente, awaiting LT and that patients with Kaiser Permanente insurance may have superior outcomes, given Kaiser Permanente’s unique integrated health care model known for its emphasis on preventive care, timely appointments for patients, and excellent care coordination.^16^ As our LT center receives referrals from various forms of public and private insurance, including serving as the referral center for all of Kaiser Permanente Northern California, we are in a key position to study this issue. Therefore, in this study, we sought to examine the association of insurance type with outcomes for patients with HCC wait-listed for LT.

## Methods

### Study Design and Patient Population

This retrospective cohort study included patients aged 18 years or older initially enrolled in a waiting list for LT at University of California, San Francisco with initial HCC Model for End-Stage Liver Disease (MELD) exceptions granted from January 1, 2010, to December 31, 2016. Patients who required tumor downstaging to be eligible according to Milan criteria for wait-listing were excluded. Collected variables included demographic characteristics (age, sex, self-reported race/ethnicity, insurance type, education level, US citizenship status, country of origin) at the time the patient was added to the LT waiting list (baseline), days between each MELD exception upgrade, size and number of tumors at the time of priority listing, number and type of local-regional therapies received, baseline α-fetoprotein (AFP) level, MELD score at baseline, cause of liver disease, and, if applicable, either reason for waiting list dropout or posttransplant histopathologic data.

This study was approved by the University of California, San Francisco Committee for Human Research. The study received expedited approval with minimal study risk assignment. The informed consent requirement was waived because this study used retrospective data without any intervention or deviation from standard of care. This report follows the Strengthening the Reporting of Observational Studies in Epidemiology (STROBE) reporting guideline.

### Outcome

The primary outcome for this study was dropout from the LT waiting list owing to liver-related death or HCC tumor progression. The secondary outcome was receipt of a deceased donor LT. Patients who underwent LT at another hospital or from a live donor (LD) were censored at the time of removal from the University of California, San Francisco waiting list, since insurance type may affect a patient’s ability to pursue these options.

### Statistical Analysis

Clinical characteristics of the groups stratified by insurance type were compared with Pearson χ^2^ and Kruskal-Wallis tests. The cumulative incidence and 95% CIs for dropout and LT were calculated while accounting for competing risks and stratified by insurance type. For the primary outcome of dropout, LT was considered a competing event. For the secondary outcome of LT, dropouts owing to liver-related death or HCC tumor progression were considered competing events.

Univariate and multivariable subdistribution hazard ratios (HRs) and 95% CIs for risk factors associated with waiting list dropout were estimated via Fine and Gray competing-risk regression. Risk factors associated with dropout with a univariate *P* value less than .10 were evaluated in the multivariable analysis. The final multivariable model was selected by backward elimination, and variables with 2-tailed *P* values of .05 or more were removed. Statistical analyses were performed with SAS statistical software version 9.4 (SAS Institute) and Stata/IC version 14.2 (StataCorp).

## Results

### Patient Characteristics

Baseline patient characteristics for the 705 patients included in the study are summarized in Table 1. For the full cohort, the median (interquartile range [IQR]) age at baseline was 61 (57-65) years, and 537 participants (76.2%) were men. There were 349 patients (49.5%) with Kaiser Permanente insurance (median [IQR] age at baseline, 61 [57-65] years; 259 [74.2%] men), 157 patients (22.3%) with other private (non–Kaiser Permanente) insurance (median [IQR] age at baseline, 61 [57-63] years; 133 [84.7%] men), and 199 patients (28.2%) with public insurance (median [IQR] age at baseline, 62 [57-66] years; 145 [72.9%] men). Median (interquartile range) follow-up was 13.2 (7.8-18.7) months. There was no statistically significant difference in age, but the difference in sex distribution was statistically significant (*P* = .02). The difference in racial/ethnic constitution was also statistically significant among insurance groups (Kaiser Permanente: 163 of 349 [46.7%] white; other private: 95 of 157 [60.5%] white; public: 73 of 199 [36.7%] white; *P* = .002). The Kaiser Permanente insurance group had the highest proportion of patients with a college or higher degree (Kaiser Permanente: 158 patients [47.9%]; other private: 65 patients [45.8%]; public: 66 patients [36.3%]; *P* < .001) and the lowest proportion of non-US citizens (Kaiser Permanente: 17 patients [5.0%]; other private: 15 patients [9.9%]; public: 34 patients [17.8%]; *P* < .001). Cause of liver disease and tumor characteristics, including AFP level, tumor size and number, and number of local-regional treatments, were similar among groups (Table 1). The Kaiser Permanente insurance group had the most patients with fewer than a median of 120 days between MELD exception upgrades (289 patients [65.3%]), followed by the other private insurance group (124 patients [79.0%]), then the public insurance group (130 patients (65.3%) (*P* < .001) (Table 2).

**Table 1.** Baseline Patient and Tumor Characteristics Stratified by Insurance Type

Variable	No. (%)				*P* Value
	Total (N = 705)	Kaiser Permanente (n = 349)	Other Private (n = 157)	Public (n = 199)	
Age, median (IQR), y	61 (57-65)	61 (57-65)	61 (57-63)	62 (57-66)	.11
Sex					
Women	168 (23.8)	90 (25.8)	24 (15.3)	54 (27.1)	.02
Men	537 (76.2)	259 (74.2)	133 (84.7)	145 (72.9)	
Education level					
Grades 1-8	48 (7.3)	15 (4.5)	5 (3.5)	28 (15.4)	<.001
High school	251 (38.4)	123 (37.3)	56 (39.4)	72 (39.6)	
Associate’s degree or college	289 (44.2)	158 (47.9)	65 (45.8)	66 (36.3)	
Graduate degree	66 (10.1)	34 (10.3)	16 (11.3)	16 (8.8)	
NR	51	19	15	17	
Race/ethnicity					
White	331 (47.0)	163 (46.7)	95 (60.5)	73 (36.7)	.002
Hispanic	153 (21.7)	70 (20.1)	27 (17.2)	56 (28.1)	
Asian	139 (19.7)	72 (20.6)	23 (14.6)	44 (22.1)	
African American	53 (7.5)	32 (9.2)	6 (3.8)	15 (7.5)	
Other	29 (4.1)	12 (3.4)	6 (3.8)	11 (5.5)	
Cause of liver disease					
Hepatitis C virus	457 (64.8)	224 (64.2)	100 (63.7)	133 (66.8)	.54
Hepatitis B virus	108 (15.3)	61 (17.5)	22 (14.0)	25 (12.6)	
Alcohol	56 (7.9)	24 (6.9)	18 (11.5)	14 (7.0)	
Nonalcoholic steatohepatitis	41 (5.8)	19 (5.4)	6 (3.8)	16 (8.0)	
Autoimmune hepatitis, primary sclerosing cholangitis, or primary biliary cirrhosis	25 (3.5)	12 (3.4)	6 (3.8)	7 (3.5)	
Other	18 (2.6)	9 (2.6)	5 (3.2)	4 (2.0)	
US-born					
Yes	309 (70.5)	153 (71.5)	74 (77.9)	82 (63.6)	.06
No	129 (29.5)	61 (28.5)	21 (22.1)	47 (36.4)	
NR	267	135	62	70	
US citizen					
Yes	616 (90.3)	322 (95.0)	137 (90.1)	157 (82.2)	<.001
No	66 (9.7)	17 (5.0)	15 (9.9)	34 (17.8)	
NR	23	10	5	8	
Blood type					
A	255 (36.2)	137 (39.3)	56 (35.7)	62 (31.2)	.48
AB	30 (4.3)	14 (4.0)	6 (3.8)	10 (5.0)	
B	84 (11.9)	39 (11.2)	23 (14.6)	22 (11.1)	
O	336 (47.7)	159 (45.6)	72 (45.9)	105 (52.8)	
MELD score, median (IQR)	10 (8-13)	10 (8-13)	10 (8-13)	11 (8-14)	.005
AFP level ≥100 ng/mL	98 (14.2)	44 (13.0)	24 (15.5)	30 (15.2)	.67
Tumor category					
1 Lesion 2-3 cm	295 (41.8)	147 (42.1)	68 (43.3)	80 (40.2)	.72
1 Lesion >3 cm	189 (26.8)	93 (26.6)	35 (22.3)	61 (30.7)	
2 Lesions	162 (23.0)	78 (22.3)	41 (26.1)	43 (21.6)	
≥3 Lesions	59 (8.4)	31 (8.9)	13 (8.3)	15 (7.5)	
Tumors, median (IQR), No.	1 (1-2)	1 (1-2)	1 (1-2)	1 (1-2)	.60
Total tumor diameter, median (IQR), cm	3.20 (2.50-4.30)	3.20 (2.50-4.40)	3.10 (2.50-4.20)	3.20 (2.40-4.40)	.98

Abbreviations: AFP, α-fetoprotein; IQR, interquartile range; MELD, Model for End-Stage Liver Disease; NR, not reported.

SI conversion factor: To convert AFP to micrograms per liter, multiply by 1.

**Table 2.** Outcomes on the LT Waiting List Stratified by Insurance Type

Outcome	No. (%)				*P* Value
	Total (N = 705)	Kaiser Permanente (n = 349)	Other Private (n = 157)	Public (n = 199)	
Time between MELD exceptions, median					
≤120 d	543 (77.0)	289 (82.8)	124 (79.0)	130 (65.3)	<.001
Only 1 exception	138 (19.6)	54 (15.5)	28 (17.8)	56 (28.1)	
>120 d	24 (3.4)	6 (1.7)	5 (3.2)	13 (6.5)	
Dropped out	246 (36.9)	100 (28.7)	53 (33.8)	93 (46.7)	<.001
Owing to tumor progression or liver-related death	174 (24.7)	67 (19.2)	41 (26.1)	66 (33.2)	<.001
Time from listing to dropout, median (IQR), mo	7.80 (3.87-14.47)	8.60 (4.63-17.03)	7.80 (3.23-13.70)	7.55 (3.80-13.73)	.50
Received deceased donor LT	416 (59.0)	229 (65.6)	99 (63.1%)	88 (44.2)	.84
Time from listing to deceased donor LT, median (IQR), mo	15.5 (9.9-18.9)	15.5 (10.1-18.6)	14.6 (9.4-19.3)	16.1 (10.7-19.3)	
Explant characteristics					
Within Milan criteria	167 (44.5)	94 (46.8)	39 (44.3)	34 (39.5)	.38
No viable tumor	122 (32.5)	67 (33.3)	30 (34.1)	25 (29.1)	
Outside Milan criteria	83 (22.1)	38 (18.9)	18 (20.5)	27 (31.4)	
Metastatic disease	3 (0.8)	2 (1.0)	1 (1.1)	0 (0)	
Microvascular invasion present	24 (6.4)	13 (6.4)	3 (3.4)	8 (9.3)	.28
Explant tumor grade					
Moderate	151 (40.1)	76 (37.6)	38 (42.7)	37 (43.0)	.47
Completely necrotic	122 (32.4)	67 (33.2)	30 (33.7)	25 (29.1)	
Well differentiated	73 (19.4)	45 (22.3)	11 (12.4)	17 (19.8)	
Poor	31 (8.2)	14 (6.9)	10 (11.2)	7 (8.1)	
Time on waiting list, median (IQR), mo	13.0 (6.5-18.2)	14.0 (7.1-18.3)	12.0 (6.2-18.3)	12.3 (5.9-18.2)	.34
Local-regional therapies, median (IQR), No.	2 (1-4)	2 (1-4)	2 (1-3)	2 (1-4)	.51
Received transplant at outside hospital	38 (5.4)	26 (7.4)	10 (6.4)	2 (1.0)	.001
Received live donor LT	20 (2.8)	10 (2.9)	5 (3.2)	5 (2.5)	.91

Abbreviations: IQR, interquartile range; LT, liver transplant; MELD, Model for End-Stage Liver Disease.

### Outcomes on the Waiting List

**Figure 1.**
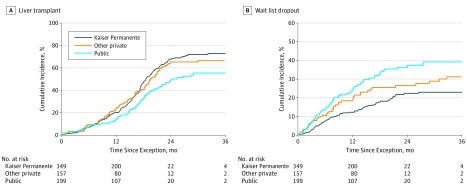
Cumulative Incidence of Liver Transplant Waiting List Outcomes by Insurance Type

Overall, 246 of 705 patients dropped out from the LT waiting list for any reason during the study. Among the full cohort, 174 of 705 patients (24.7%) dropped out from the LT waiting list owing to tumor progression or death. This included 67 of 349 patients with Kaiser Permanente insurance (19.2%), 41 of 157 patients with other private insurance (26.1%), and 66 of 199 patients with public insurance (33.2%) (Table 2). The cumulative incidence of dropout owing to tumor progression or death within 1 year of baseline was 16.5% overall and 26.4% overall within 2 years of baseline. At 1 year, the cumulative incidence of dropout was 11.9% (95% CI, 8.7%-15.7%) among patients with Kaiser Permanente insurance, 17.6% (95% CI, 12.0%-24.2%) among patients with other private insurance, and 23.6% (95% CI, 17.8%-29.9%) among patients with public insurance. At 2 years, the cumulative incidence of dropout was 21.8% (95% CI, 17.2%-26.7%) among patients with Kaiser Permanente insurance, 25.5% (95% CI, 18.6%-33.0%) among patients with other private insurance, and 35.5% (95% CI, 28.3%-42.7%) among patients with public insurance (Kaiser Permanente insurance vs public insurance: *P* < .001) (Figure 1A). Median (IQR) time to dropout was 7.8 (3.9-14.5) months overall and did not differ by insurance type (Table 2).

**Figure 2.**
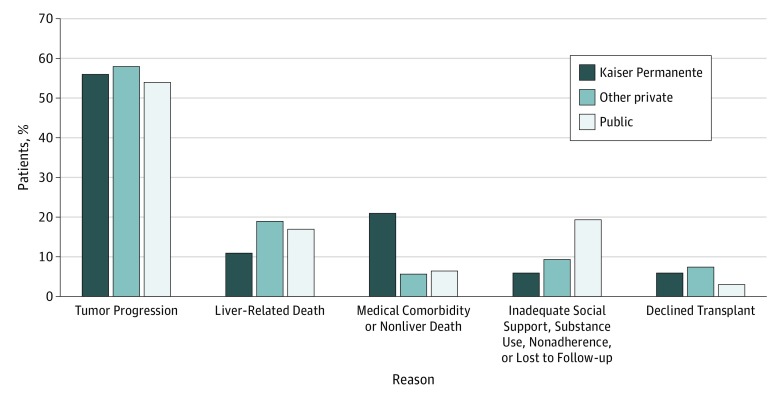
Liver Transplant Waiting List Dropout Stratified by Insurance Type and Reason for Dropout

An additional 72 of 705 patients (10.2%) in the full cohort dropped out from the LT waiting list for reasons other than tumor progression or liver-related death. Thirty patients (4.3%) dropped out owing to another medical comorbidity or non–liver-related death, and 29 patients (4.1%) dropped out because they had inadequate social support, had substance use disorder, were nonadherent, or were lost to follow-up. Nineteen patients (2.7%) transitioned to a different insurance type after being added to the LT waiting list with HCC MELD exception but before their wait-list outcome. Thirteen patients (1.8%) declined LT (Figure 2). Among the Kaiser Permanente insurance group, 6 patients (6.0%) dropped off the waiting list because of inadequate social support, substance use, nonadherence, or being lost to follow-up, compared with 5 patients with other private insurance (9.4%) and 18 patients with public insurance (19.4%).

In the full cohort, 416 of 705 patients (59.0%) underwent deceased donor LT during the study, including 229 of 349 patients with Kaiser Permanente insurance (65.6%), 99 of 157 patients with other private insurance (63.1%), and 88 of 199 patients with public insurance (44.2%). The cumulative incidence of deceased donor LT was 18.6% overall at 1 year from baseline and 61.3% at 2 years from baseline. At 2 years from baseline with HCC MELD exception, the cumulative incidence of deceased donor LT was 67.3% (95% CI, 61.2%-72.6%) for patients with Kaiser Permanente insurance, 64.1% (95% CI, 55.2%-71.7%) for patients with other private insurance (*P* = .72), and 48.5% (95% CI, 40.4%-56.1%) for patients with public insurance (*P* < .001) (Figure 1B). Median (IQR) time to LT was 15.5 (9.9-18.9) months overall and did not differ by insurance type (*P* = .84). There was no statistically significant difference in explant histopathologic stage, microvascular invasion, or tumor differentiation among groups (Table 2). At the end of the study, 43 patients (6.1%) were still active on the LT waiting list.

Overall, 58 patients (8.2%) underwent LT outside of our study, either by LT at another hospital and United Network for Organ Sharing region (38 patients [5.4%]) or by LDLT (20 patients [2.8%]). Only 7 of these 58 patients (12.1%) had public insurance, 2 of whom received LT at another hospital and 5 of whom underwent LDLT. Thirty-six of these 58 patients (62.1%) had Kaiser Permanente insurance, 26 of whom received LT at another hospital and 10 of whom underwent LDLT (Table 2).

### Factors Associated With Waiting List Dropout

In univariate competing-risk regression, statistically significant factors associated with LT waiting list dropout were having public insurance (HR vs Kaiser Permanente insurance, 1.93 [95% CI, 1.38-2.71]; *P* < .001), AFP level of 100 ng/mL (to convert to micrograms per liter, multiply by 1) or higher (HR vs <100 ng/mL, 2.57 [95% CI, 1.84-3.58]; *P* < .001) at baseline, MELD score at baseline (HR per point, 1.06 [95% CI, 1.03-1.16]; *P* < .001), 3 or more HCC lesions at baseline (HR vs 1 lesion with 2- to 3-cm diameter, 2.23 [95% CI, 1.38-3.61]; *P* < .001), and a median of more than 120 number of days between MELD exception upgrades (HR vs ≤120 days, 0.20 [95% CI, 0.06-0.62]; *P* = .005) (Table 3). Variables that were not statistically significantly associated with waiting list dropout included age, sex, education level, race/ethnicity, type of liver disease, blood type, and US citizenship status (Table 3).

**Table 3.** Univariate and Multivariate Analysis of Waiting List Dropout Owing to Tumor Progression or Death

Characteristic	Hazard Ratio (95% CI)	*P* Value
**Univariate Analysis**		
Insurance type		
Kaiser Permanente	1 [Reference]	NA
Other private	1.42 (0.97-2.10)	.07
Public	1.93 (1.38-2.71)	<.001
Age at baseline, per y	1.01 (0.99-1.03)	.29
Women^a^	1.14 (0.81-1.59)	.46
Education level		
Grades 1-8	1 [Reference]	NA
High school	1.22 (0.66-2.26)	.53
Associate’s degree or college	1.12 (0.61-2.07)	.72
Graduate degree	0.92 (0.41-2.02)	.83
Race/ethnicity		
White	1 [Reference]	NA
Hispanic	1.02 (0.69-1.50)	.94
Asian	0.73 (0.47-1.12)	.15
African American	1.56 (0.91-2.56)	.11
Other	2.56 (1.52-4.32)	<.001
Cause of liver disease		
Hepatitis C virus	1 [Reference]	NA
Hepatitis B virus	0.70 (0.44-1.12)	.14
Alcohol	1.21 (0.68-2.13)	.51
Nonalcoholic steatohepatitis	0.94 (0.49-1.80)	.84
Autoimmune hepatitis, primary sclerosing cholangitis, or primary biliary cirrhosis	1.22 (0.51-2.94)	.65
Other	0.77 (0.32-1.86)	.56
Blood type		
A	1 [Reference]	NA
AB	0.48 (0.17-1.35)	.17
B	1.04 (0.63-1.72)	.87
O	1.00 (0.72-1.38)	.99
AFP level ≥100 ng/mL^b^	2.57 (1.84-3.58)	<.001
US citizen	0.98 (0.59-1.63)	.93
US-born	1.07 (0.70-1.62)	.76
MELD score at baseline, per point	1.06 (1.03-1.16)	<.001
Tumor category		
1 Lesion 2- to 3-cm diameter	1 [Reference]	NA
1 Lesion >3-cm diameter	1.39 (0.97-2.01)	.08
2 Lesions	1.22 (0.81-1.84)	.33
≥3 Lesions	2.23 (1.38-3.61)	.001
No. of local-regional therapies	1.11 (1.04-1.19)	.003
Time between MELD exceptions, median		
≤120 d	1 [Reference]	NA
Only 1 exception	0.24 (0.17-0.33)	<.001
>120 d	0.20 (0.06-0.62)	.005
**Multivariate Analysis**		
Insurance type		
Kaiser Permanente	1 [Reference]	NA
Other private	1.40 (0.94-2.08)	.10
Public	1.69 (1.17-2.43)	.005
AFP level ≥100 ng/mL^b^	2.77 (1.98-3.88)	<.001
MELD score	1.06 (1.03-1.09)	<.001
Tumor category		
1 Lesion 2- to 3-cm diameter	1 [Reference]	NA
1 Lesion >3-cm diameter	1.24 (0.85-1.83)	.26
2 Lesions	1.28 (0.84-1.94)	.25
≥3 Lesions	2.07 (1.27-3.37)	.004

Abbreviations: AFP, α-fetoprotein; MELD, Model for End-Stage Liver Disease; NA, not applicable.

SI conversion factor: To convert AFP to micrograms per liter, multiply by 1.

^a^ Compared with men.

^b^ Compared with AFP level less than 100 ng/mL.

On multivariable analysis, compared with patients with Kaiser Permanente insurance, the risk of dropout was increased for patients with public insurance (HR, 1.69 [95% CI, 1.17-2.43]; *P* = .005). For patients with other private insurance, the difference was not statistically significant (HR, 1.40 [95% CI, 0.94-2.08]; *P* = .10). Risk of dropout for patients with public insurance was similar compared with those with other private insurance on multivariable analysis. Waiting list dropout was significantly associated with an AFP level of 100 ng/mL or higher (HR, 2.77 [95% CI, 1.98-3.88]; *P* < .001), MELD score at baseline (HR per point, 1.06 [95% CI, 1.03-1.09]; *P* < .001), and 3 lesions at baseline (HR vs 1 lesion with 2- to 3-cm diameter, 2.07 [95% CI, 1.27-3.37]; *P* = .004) (Table 3).

## Discussion

Liver transplant is the best curative therapy for patients with nonresectable HCC, yet the scarcity of organs and resulting long waiting list times leads to significant risk of dropout owing to cancer progression or hepatic decompensation. Dropout rates from the LT waiting list are relatively high, approximately 15% to 30% at 1 year from baseline.^15^ Several patient characteristics are associated with increased risk of waiting list dropout for patients with HCC, including MELD score at baseline,^17,18,19^ the presence of multifocal HCC, larger tumor size, and high peak AFP level.^20,21,22^ While these liver- and tumor-related variables are clearly associated with wait-list outcomes, to our knowledge, no prior studies have specifically examined the association of insurance type on outcomes on the LT waiting list. In this study, we found that patients with public insurance were associated with worse wait-list outcomes and increased risk of dropout owing to tumor progression or liver-related death compared with patients with private insurance, including Kaiser Permanente insurance, despite similar tumor- and liver disease–related characteristics. Specifically, having public insurance was associated with a nearly 70% increased risk of dropout compared with having Kaiser Permanente insurance (HR, 1.69 [95% CI, 1.17-2.43]).

Variations in the management of HCC depending on insurance type and socioeconomic status have been well documented over the past few decades. In 2006, a large population-based study^23^ that included 2963 predominantly Medicare patients with HCC diagnosed between 1992 and 1999 found that most patients did not receive potentially curative therapy and only one-third of patients with favorable tumor characteristics who were likely to benefit from surgical treatment received such therapy. More recently, a 2018 study^9^ examining 32 388 patients with HCC diagnosed between 2007 and 2012 found that patients who were uninsured or had Medicaid had more advanced tumor stage at diagnosis, lower rates of treatment, and lower overall survival.

Importantly, our study found that, despite similar tumor- and liver disease–related characteristics at the time of listing with an HCC MELD exception, patients with public insurance were the least likely to undergo LT and had the highest rate of waiting list dropout compared with patients with Kaiser Permanente or other private insurance. The public insurance group had the smallest proportion of patients with higher education, the most non-US citizens, and the most nonwhite patients. Public insurance remained statistically significantly associated with waiting list dropout compared with Kaiser Permanente and other private insurance even after controlling for other factors associated with waiting list dropout, including AFP level, MELD score at baseline, and number of HCC lesions. Our study also found that patients with public insurance had significantly longer periods between MELD exception upgrades compared with patients with Kaiser Permanente or other private insurance. Furthermore, only 5% of patients who underwent LT at another health care center had public insurance compared with 68% of patients who had Kaiser Permanente insurance.

Although there has been much speculation, the reasons for discrepancy in care between patients with private and public insurance remain unclear, to our knowledge. Explanations previously put forth include that patients with public insurance present with more advanced disease than those with private insurance^24^ and that Medicaid patients generally have less access to subspecialty care.^25^ Yet, one 2015 study^26^ showed that mortality and survival were worse for patients with public insurance than for those with private insurance even when presenting with early-stage HCC. In our study, all patients had similar tumor-related characteristics and had accessed subspecialty care, as demonstrated by listing on the LT waiting list; therefore, there must be other factors to explain our findings aside from tumor stage and ability to access subspecialty care.

Patients with Medicaid are more likely to be poor and unemployed and to have less education and more comorbidities than patients with private insurance,^27^ all of which may contribute to worse wait-list outcomes. Lack of stable housing and adequate social support could negatively affect a patient’s ability to attend health care appointments and increase the likelihood of being lost to follow-up. Lower health care literacy, which could result in a lack of understanding of disease severity and the importance of attending appointments, is also significantly associated with lower education levels and with language barriers.^28^ These factors may have contributed to our finding that patients with public insurance had significantly longer periods between MELD exception upgrades. In our study, 19% of patients who dropped out of the waiting list from the public insurance group did so because of inadequate social support, nonadherence, or being lost to follow-up, compared with 6.0% of patients from Kaiser Permanente insurance and 9.4% of patients with other private insurance, supporting the hypothesis that socioeconomic factors may contribute to increased waiting list dropout among patients with public insurance.

Our study did not show a significant difference in risk of waiting list dropout between patients with public insurance compared with patients with other private insurance. The other private insurance cohort was relatively small and may have been underpowered to detect significant differences between these groups. Additionally, since most other private insurance groups have no specific care coordination or navigation for patients with HCC awaiting LT, we believe that this feature, which is somewhat unique to the Kaiser Permanente model, may be associated with the improved wait-list outcomes seen in the Kaiser Permanente insurance cohort. If the major driver of worse wait-list outcomes (aside from tumor characteristics and severity of liver disease) had been associated with socioeconomic factors, we would have expected patients with Kaiser Permanente insurance or other private insurance to have similarly improved outcomes compared with patients with public insurance, but this is not what was observed in this study.

Additional components of waiting list management for patients with HCC include referring to other centers with shorter wait times and counseling on LDLT as an alternative to deceased donor LT. Our study found that only a very small proportion of patients who received LT at another center or LDLT had public insurance. Increased discussion of these options between the health care team and patients could help address these particular issues. Additionally, in May 2019, United Network for Organ Sharing enacted policy assigning HCC exception points based on median MELD at transplant minus 3 points (called *MMaT-3*).^29^ With this change, the incentive for patients with private insurance to travel to other regions may disappear, which could help reduce some of these disparities, since patients with public insurance typically do not have this option.

### Strengths and Limitations

Our study has several strengths. Most notably, this is the first study evaluating outcomes for patients with HCC on the LT waiting list by insurance type, to our knowledge. We had a large patient cohort of more 700 patients and representation of 3 different insurance types at our transplant center. Given the long wait-list time in our region, we are in a prime position to study risk factors associated with waiting list dropout.

Our study also had limitations. Limitations to our study included its retrospective design and the fact that it was a single-center study. Our patient population in Northern California was relatively diverse; therefore, there may be issues with extrapolating our results to other centers with more homogeneous patient populations. Additionally, less than 3% of patients in our cohort transitioned to a different insurance type, so we were unable to include an analysis of insurance transition in our study. Furthermore, the specific reasons for longer MELD exception periods for patients with public insurance, which may have contributed to increased dropout rate in this patient cohort, remain unclear, and we were not able to investigate this variable given the retrospective nature of this study.

## Conclusions

In conclusion, in this large, diverse cohort of patients with HCC on a waiting list for LT, patients with public medical insurance had worse wait-list outcomes despite similar tumor- and liver disease–related characteristics compared with patients with Kaiser Permanente medical insurance or other private medical insurance. These findings appear to be associated with delays in completing pre-LT evaluations and obtaining timely MELD exception upgrades. Public insurance should be recognized as a risk factor associated with waiting list dropout, and necessary steps should be implemented to mitigate the increased risk of dropout among these patients. These findings have increasingly meaningful implications as more patients are using public insurance to pay for LT.^13,14^ Improved health care coordination and delivery could potentially help to reduce these disparities.
